# Phototriggerable Liposomes: Current Research and Future Perspectives

**DOI:** 10.3390/pharmaceutics6010001

**Published:** 2013-12-19

**Authors:** Anu Puri

**Affiliations:** Membrane Structure and Function Section, Basic Research Lab, Center for Cancer Research, National Cancer Institute at Frederick, National Institutes of Health, Bldg 469/Rm 216A, 1050 Boyles Street, Frederick, MD 21702-1201, USA; E-Mail: puria@mail.nih.gov; Tel.: +1-301-846-5069; Fax: +1-301-846-6210

**Keywords:** lipid-based nanoparticles, drug delivery, laser, cancer therapy, photodynamic therapy, liposomes, phototriggering, cancer nanomedicine

## Abstract

The field of cancer nanomedicine is considered a promising area for improved delivery of bioactive molecules including drugs, pharmaceutical agents and nucleic acids. Among these, drug delivery technology has made discernible progress in recent years and the areas that warrant further focus and consideration towards technological developments have also been recognized. Development of viable methods for on-demand spatial and temporal release of entrapped drugs from the nanocarriers is an arena that is likely to enhance the clinical suitability of drug-loaded nanocarriers. One such approach, which utilizes light as the external stimulus to disrupt and/or destabilize drug-loaded nanoparticles, will be the discussion platform of this article. Although several phototriggerable nanocarriers are currently under development, I will limit this review to the phototriggerable liposomes that have demonstrated promise in the cell culture systems at least (but not the last). The topics covered in this review include (i) a brief summary of various phototriggerable nanocarriers; (ii) an overview of the application of liposomes to deliver payload of photosensitizers and associated technologies; (iii) the design considerations of photoactivable lipid molecules and the chemical considerations and mechanisms of phototriggering of liposomal lipids; (iv) limitations and future directions for *in vivo*, clinically viable triggered drug delivery approaches and potential novel photoactivation strategies will be discussed.

## 1. Introduction

### 1.1. Nano Drug Delivery Systems

Nanoparticulate systems based on unique lipid assemblies have been long sought to improve delivery of anticancer agents and some platforms, primarily liposomes are currently in use for patient care [1,2,3,4,5,6,7]. Furthermore, these delivery systems coupled with site-specific targeting ligands constitute the potential to boost efficacy and bioavailability of existing drugs and pharmaceuticals [1,3,8,9,10,11]. Optimal drug delivery systems feature multifunctional nanoparticles with imaging molecules, a pay-load of drugs, targeting ligands, destabilization elements as well as sensors that probe the efficacy of the drug in real time [12,13,14,15,16,17]. Some widely examined nanocarriers aimed at delivering nucleic acids, pharmaceuticals and/or imaging agents include dendrimers [18,19], nano-gold shells [20], nano-emulsions [21], drug-polymer conjugates [22,23,24], drug-antibody conjugates [25], quantum dots [26,27,28], aptamer-gated nanovehicles [29], and solid lipid nanoparticles [30]. Each of these nanotechnology platforms entails unique fabrication components that rely on self-assembly of the structural motifs of the building blocks of the particles, while accommodating the pharmaceutical agent and/or the targeting ligand. Recent progress in the area of theranostics medicine (combining therapy and diagnostics) is likely to impact the outcome of success in the nanomedicine field [31,32,33,34,35,36]. Liposomes consisting primarily of phospholipid assemblies, offer the advantage of being constructed from biocompatible molecules, with efficient drug loading capacity, targeting potentials and tunable on-demand drug release properties. 

### 1.2. Light-Guided Therapies, General Considerations

The success of light-guided therapy is dependent on the choice of adequate light sources that can penetrate the tissues for drug delivery and therapeutic applications. The preferred choice of wavelengths is in the near-infrared range (700 nm to 2500 nm) as the light penetration is more than 1 cm depth into human skin and blood [37]. Wavelength sources below 700 nm are considered to have poor penetration deeply into tissues due to the scattering and presence of endogenous light absorbers, such as oxy- and deoxy-hemoglobin, lipids and water [38]. To obviate the tissue-penetration concerns, light-guided therapy technologies have been more widely used to areas such as skin and/or oral cavity as well for oral treatments [39,40]. In lieu of the tissue penetration limitation, and currently available light guides, phototriggerable therapies are likely to succeed in the treatment of diseases such as bladder and colon cancer. To develop clinically suitable liposomes, the choice of disease for treatment will be an important factor for consideration for success of phototriggerable liposomes. 

## 2. Liposomes as Drug Delivery Platforms: An Overview

**Figure 1:**
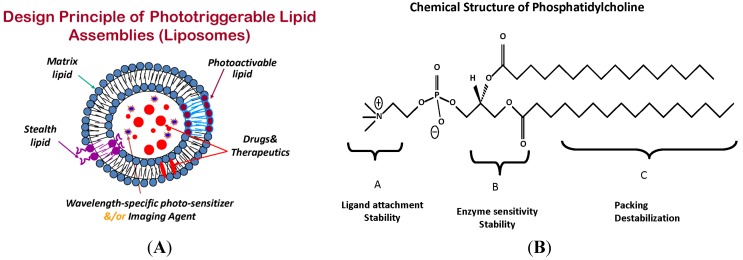
(**A**) Phototriggerable liposome assembly. Liposomes are prepared from a matrix (bulk) lipid (grey), with embedded photoactivable lipid (brown). A pegylated lipid is included to achieve stealth properties (purple). Drugs (red), imaging agents and/or a second photosensitizer (bright blue) is encapsulated in the liposomes; (**B**) Sites for Chemical modifications in phospholipids. The chemical structure of phosphatidylcholine is shown as prototype. Three major parts of phospholipids that can be chemically modified to generate photosensitive molecules. The lipid parts: head group, glycerol backbone and fatty acyl chains are described with their proposed modifications.

Among other drug carriers for cancer treatment, liposomes are the longest-studied nanoparticles and are hence associated with a number of historic milestones including development of stealth liposomes and efficient drug loading by ammonium sulfate gradient protocol [27,41,42,43,44,45,46,47,48,49]. Liposomes primarily consist of phospholipids, the major components of biological membranes [50]. Phospholipids, being natural ingredients, are considered relatively non-toxic and with their degradation by various enzymes present in the body (Figure 1A, liposomes and Figure 1B, phosphatidylcholine structure). Doxil/caylex (a liposome-based formulation of an anticancer drug Doxorubicin, Ben Venue Laboratories, Bedford, OH, USA) was the first formulation approved for its application in the clinic [51]. Important landmarks that led to the success in the liposome field have resulted from several breakthroughs including the utilization of pegylated lipids to overcome liver and spleen accumulation [52,53], and remote drug loading protocols to achieve payload of week bases in the interior of liposomes [49]. A number of liposome formulations are currently used in the clinic while others in the pipeline await clinical trials [15]. Liposomes bearing specific ligands for site-specific drug delivery (targeted liposomes) have been examined for years by using various targeting ligands from small molecules such as peptides to affibodies and antibodies. However, the clinical benefits associated with targeting remain to be seen in a clinical setting. 

It can be envisioned that once the drug-loaded particles have reached their desired site, the kinetics and extent of drug release from targeted nanoparticles will play a significant role in the outcome of disease treatment. The site of drug delivery (intra- or extra-cellular) in the tumor area can also be considered another important determinant of efficacy of drug action and overall treatment outcome. Therefore, development of tunable liposomes (as well as other nanoparticles) containing switches for *on-demand drug release* (*Triggering*) are the subject of current and future considerations to attain better therapeutic index of encapsulated drugs. 

## 3. Triggerable Liposomes

**Figure 2:**
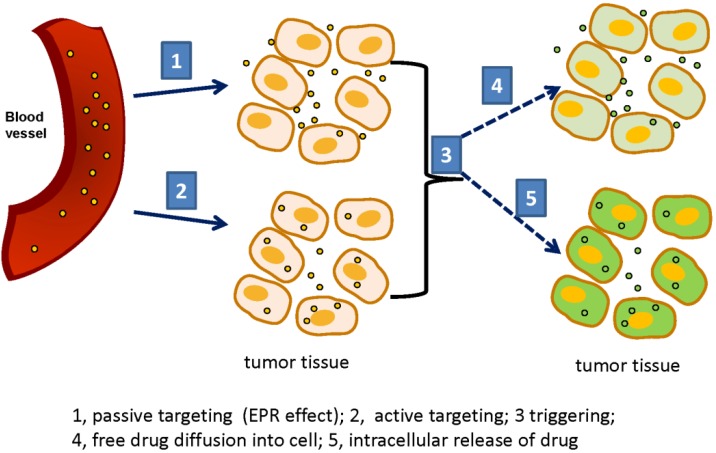
Liposomal nanomedicine: membrane barriers. The cartoon shows various membrane barriers in liposomal nanomedicine. Drug loaded liposomes (yellow circles) loaded with, calcein, a water soluble fluorescent molecule as a model drug is shown here. At high concentration calcein is quenched and is non-fluorescent (indicated by yellow color). Upon release from liposomes, calcein becomes fluorescent 9 indicated by green color). Liposomes intravenously injected into animal cross the blood vessel (red) and accumulate in the tumor area by the EPR effect (**1**, passive targeting). Targeted liposomes are taken up by the cells (**2**, active targeting). Upon triggering (**3**), drug is released from liposomes. Passively targeted liposomes release drug in the vicinity of tumor cells and then drug is taken by the cells by passive diffusion (**4**, shown by light green-indicating low effective concentration of the drug). Actively targeted liposomes release their cargo intracellularly (**5**, bright green, showing efficient drug delivery).

To date, various triggering modalities for selective release of drugs from liposomes (once they have reached their target site) have been developed and can be broadly classified into two categories namely internal and external triggers [2,14,54]. Internal triggering typically includes either exploitation of low pH in the endosome, use of enzymes overexpressed in diseased states and/or modulation of redox-potential in the liposomes. In contrast, external triggering system, as the name indicates utilizes outside forces such as heat, light or magnetic field for on-site disruption of liposomes. The principles underlying improved drug delivery upon external triggering in shown in Figure 2 (cartoon). Drug-loaded liposomes accumulate in the tumor area (circles yellow, in its concentrated form) by Enhanced Permeability and Retention effect (EPR effect, Figure 2, steps 1 and 2). Ligand-bearing particles are taken up by the cells via receptor-mediated endocytosis (Figure 2, step 2). Upon treatment with external stimuli (such as heat or light) at the tumor site (Figure 2, step 3), liposomes release their cargo (indicated by green), now diluted in a larger volume. For non-targeted liposomes, drug is released in the extracellular matrix of the tumor and then taken up by passive diffusion into the cells (Figure 2, step 4) shown by light green, indicating limited uptake of drug. In contrast, once liposomes are internalized, a payload of drug is released intracellular upon triggering (Figure 2, step 5) indicated by bright green showing larger concentration of the drug. It should be borne in mind that various triggering modalities may also have either direct or indirect effects on the tumor biology and on the treatment outcome. 

Thermosensitive liposomes first described in late 1970s have been examined for their suitability in Phase III clinical trials. Thermosensitive liposomes are based on the formation of phase boundaries (local defects) in the lipids bilayer at the phase transition of the lipids. The lipid of choice in these liposomes is DPPC (*T*m 41 °C) along with other pore forming lipids (such as lysolipids). These liposomes are thus far the most studied example of triggerable nanoparticles [55,56,57,58,59,60,61]. Thermosensitive formulation, ThermoDox^®^ (Celsion Corporation, Lawrenceville, NJ, USA) was developed for treatment of various cancers including primary liver cancer (HCC), recurrent chest wall (RCW) breast cancer (DIGNITY study), colorectal, pancreatic and metastatic liver cancer. The treatment protocols also include either radiofrequency ablation (RFA) and/or high intensity focused ultrasound (HIFU) in combination with ThermoDox^®^. The outcome of HEAT study for HCC using the ThermoDox^®^ (Phase III clinical trial) was not expected and further analysis may shed light on the modification of treatment modules such as RFA treatments, *etc.* (Celsion Corporation, Lawrenceville, NJ, USA). However, the efforts using the ThermoDox^®^ are continued for other cancer types and the results are awaited. An alternate modality to destabilize thermosensitive liposomes relies on laser-induced disruption to release the cargo. Mackanos *et al.* reported *in vivo* disruption of thermosensitive liposomes by using a Nd:YLF laser (527 nm) by monitoring luciferin release [62]. Thermodox^®^ contains lysoPC as one of the molecules to modulate temperature-triggered destabilization of liposomes. Recently, Tagami *et al.* have described Brij78-liposomes as an alternate robust thermosensitive formulation [63]. Inclusion of surfactants to modulate thermosensitiviy is an interesting concept and has the potential to be applied to other systems as well. Another external triggering system in the field of nanomedicine is based on the exploitation of the magnetic properties superparamagnetic iron oxide particles (SPION) [64]. Attempts have been made to include these particles into liposomes along with the drug of choice, and this system bears the advantage of image-guided drug delivery [64,65,66,67]. 

## 4. Phototriggerable Nano Drug Delivery Platforms

The success of light-triggered drug delivery relies on a series of factors including phototriggerable building blocks of a nanoparticles, photosensitizing properties of the drugs being tested, appropriate light source and patient-friendly light delivering guide (instrumentation) [13,16,68,69,70]. Pharmaceutical agents typically called as photodynamic drugs or photosensitizer (PS) are light-sensitive and promising candidates for photodynamic therapy (PDT) [71,72,73]. Since these drugs are generally hydrophobic, *in vivo* delivery and efficacy is improved by nano-particulate formulations including conventional liposomes (here the lipid core is not sensitive to light *per se*) [39,74,75]. Non-phototriggerable liposome formulations containing the PDT drugs have been examined in clinical trials and therefore basic technical information (such as light sources, dosage, skin toxicities, *etc.*) from these studies will have an advantage for the development of phototriggerable liposomes as well as other light-sensitive nanoparticles. A successful example of PS-liposome formulation is Visudyne therapy that is currently clinically used. This platform is discussed later in this article. 

Currently, a number of novel and unique photosensitive nanoplatforms for imaging and PDT are being developed including the liposome-based systems (Table 1) [76,77]. Recent work by Chen and colleagues describing metal-ion based light-triggered theranostics using carbon dots (C-dots) [78,79] and PS-functionalized gold nanostars offers interesting possibilities for potential future candidates for light-triggered drug delivery [80]. The same group has also reported Ce6-loaded gold vesicles with the propensity of trimodal effect including photo-thermal and photodynamic therapy (PTT/PDT) [81]. Another light-triggerable platform described by Lapotko and colleagues relies on the utilization of plasmonic nanobubbles to disrupt liposomes and release drugs [82]. Here, the gold nanoparticles are loaded into liposomes and exposed to short laser pulses to produce transient vapor bubbles causing disruption. The process appears to be mechanical in nature and not triggered by heat. The *in vivo* demonstration of this technology was done using the zebrafish and the further application in mammalian models is awaited. Recently, studies describing another interesting platform called “light-responsive polymer nanoreactors” for on demand production of ROS were reported [83,84]. The polymeric nanoparticles loaded with a photosensitizer-protein conjugate, can be triggered with an appropriate light source generating ROS as desired. Another viable photosensitive polymer design described by Almutairi and colleagues consists of multiple photo-activatable groups along with inclusion of a quione-methide self-immolative group [85,86]. These particles were phototriggerable by the near-IR light source and therefore may find future for *in vivo* applications. Another phototriggerable platform includes dendrimer-phthalocyanine [87,88]. It is beyond the scope of this review to provide a thorough coverage of these systems; therefore, I will restrict myself to liposome-based systems with prime focus on the liposomes that have been test at least in the cell-based systems. I will provide a brief overview of conventional liposome formulations that are in works to deliver various photosensitizers at high concentrations to the site of disease (photodynamic therapy, PDT). The main part of this review will deal with the design principles of photo-triggerable lipids and molecular mechanisms that result in photo-destabilization of liposome membrane. Lastly, I will present my view on opportunities that may lead to a successful application of phototriggerable liposomes in the clinic. 

**Table 1:** Partial list of photo-sensitive nanoparticulate formulations and phototriggerable platforms.

Platform	Photo-sensitive component agent	Objective	Current status	Reference
liposomes	verteporfin (laser 689 nm)	delivery of photosensitizer	in clinic (visudyne)	[89]
liposomes	lipids/nanogold laser-(photothermal)	triggered drug delivery	*in vitro* and animal studies	[62,77]
gold nanostars/vesicles	photosensitizers (clorin e6)	drug delivery PTT/PDT	*in vitro*/*in vivo*	[80,81]
carbon dots	metal ions	theranostics	*in vitro*/*in vivo*	[78,79]
plasmonic nanobubbles	mechanical by laser	drug delivery	*in vitro*/*in vivo*	[82]
polymers	photoprins phthalocyanines	neovascularization	*in vitro*/*in vivo* studies	[85,86]
polymers nanoreactors	photosensitizer	ROS production	*in vitro*	[83,84]
dendrimers polymeric micelles	porphyrin phthalocyanine	drug delivery	*in vitro*	[87,88]

## 5. Photodynamic Drug-Liposome Formulations

Photodynamic therapy (PDT) is generally based on light-mediated activation of a photosensitizing molecule resulting in generation of reactive oxygen species (ROS) and destruction of target cells and tissues. Photoactivation of photosensitizing drugs (PDT drugs) occurs via distinct mechanisms (Type I or Type II) and the photochemistry of various PDT drugs has been investigated in details [72,73,90,91,92,93,94,95,96,97]. A general mechanism by which a PDT drug exerts its action upon light activation involves absorption of photons followed by a triplet state excitation. This step then leads to either generation of ROS (Examples: superoxide anion, hydroxyl radical, hydrogen peroxide) or transfer of energy to a ground-state molecular oxygen followed by production of a highly reactive singlet oxygen. The classes of PDT drugs include porphyrin derivatives, chlorins, phthalocyanines, and porphycenes [69,92,98]. Some clinical attempts to improve treatment of cancers and infectious diseases by PDT include non-respectable hilar bile duct cancer (drug used temoprfin) [99], oral cancer (photofrin, liposomal aluminum-cholride phthalocyanine) [100], pain determination in patients (red light/5-aminolevulinic acid) [93,101,102]. Readers are also referred to a recent review by Mamalis *et al.* for the laser and light treatments of keloids [103]. 

Despite advances made by clinically available photosensitizing agents, full potential of these agents has not been achieved. Due to their hydrophobic properties, photosensitizers meet technical challenges of being poorly soluble and their propensity to aggregate in aqueous phases and hence their limited delivery in active form to the desired cite [104,105]. Additionally, an inadequate affinity by most photosensitizers to tumor sites also results in some damage of normal tissue following PDT in patients. Nanotechnology based formulations of photosensitizers are attractive systems for improved delivery of photosensitizers [39,74,75]. 

### Visudyne Therapy

Although a number of liposome formulations have been developed since decades to deliver photosensitizers for PDT for cancer treatment, a successful application thus far is Visudyne therapy [72,106,107,108,109]. Visudyne^®^ (MedKoo Biosciences, Inc., Chapel Hill, NC, USA) is a liposome formulation containing verteporfin (BPD-MA) as the PDT drug and this formulation contains BPD-MA:Egg phosphatidyl glycerol: dimyristoyl PC at the molar ratios of 1:05:3:5 of these components respectively. The formulation also contains ascorbyl palmitate, butylated hydroxytoluene and lactose as additional inactive ingredients. This formulation is used to treat age-related macular degeneration of (AMD) in patients. The treatment course is intravenous injection of Visudyne followed by non-photothermal treatment with a 689 nm laser source. More information about the doses, light treatment conditions, types of lasers approved and side effects *etc.* can be found at http://www.visudyne.com/ [89]. Clinical advantage of Visudyne for the treatment of cancer still remains to be seen. 

## 6. Phototriggerable Liposomes-Background

Liposomes constituted from light-sensitive lipids have been explored since early 1980s. As discussed above, the thermo-sensitive liposomes rely on the principle of phase transition properties of the phospholipids. In contrast, typically a phototriggerable liposome system includes a light-sensitive group chemically engineered into the lipid of choice. The overall goal of using triggerable lipids is introduce defects in the liposome membrane for localized drug delivery [2,69]. The potential sites for modification within the phospholipid molecule can be divided into three regions, namely, head group, glycerol backbone and fatty acyl chains (see Figure 1A). The fatty acyl modifications have been thus far the prime focus to generate phototriggerable liposomes with the exception of head-group polymerizable lipids. Since fatty acyl chain length and degree of unsaturation are the major determinants for lipid packing, this region presents opportunities to tune the phototriggering as desired [110]. 

**Figure 3:**
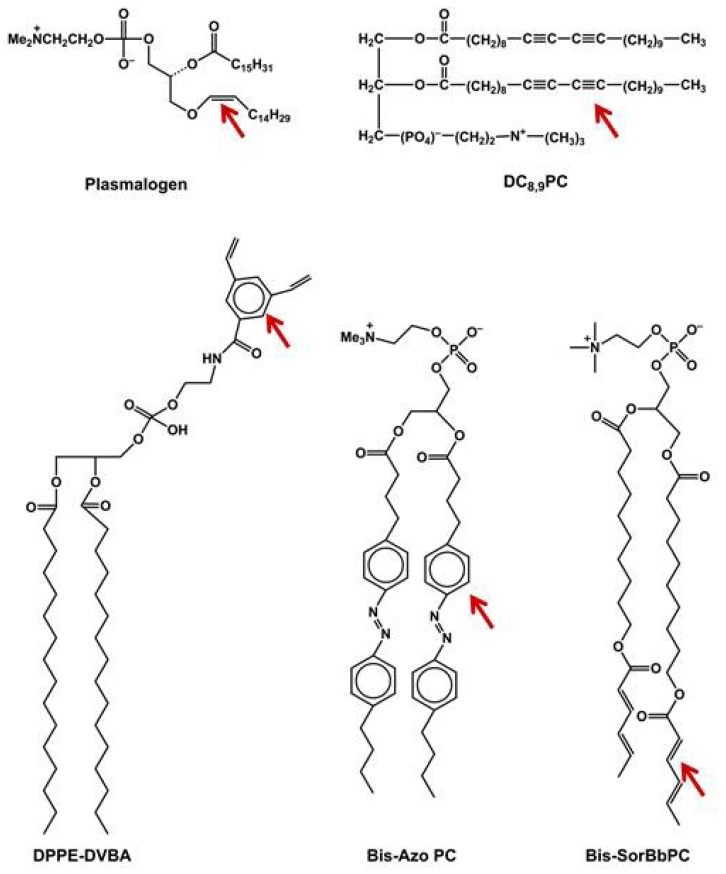
Phototriggerable Lipids: some examples. A partial list of phototriggerable lipids studied to date. The photoactivable groups are indicated by arrows.

A partial list of currently available photoactivable lipids is shown in Figure 3. The reader is referred to previous reviews for further details on the structure-function relationship of photoactivable groups in the modified lipids. Although the majority of light-sensitive lipids examined thus far have been chemically synthesized, one exception is plasmalogen (Figure 3, top left). Plasmalogen is a naturally occurring ether phospholipid, found in abundance in tissues such as heart and brain. This lipid has characteristic vinyl ether linkage at the *sn*-1 position and the ester linkage at the *sn*-2 position. It has been proposed to protect cells against damage by ROS, and signaling events. In early 1990s, Thompson and colleagues exploited the reactivity of these vinyl ether linkages (bearing at least one double bond) to develop phototriggerable liposomes [68]. These studies included specific photosensitizers such as Zn-phthalocyanine, octabutoxyphthalocyanine, and bacteriochlorophyll-α to generate ROS and react with the vinyl ether linkages. The resulting lyso-product was the initiator of destabilization of the liposomes. 

Lysolipids are generally considered pore-forming lipids and were previously included in thermosensitive liposomes to enhance temperature-triggered destabilization [55,57]. The introduction of a photosensitive moiety in the fatty acyl chains of phospholipids yields light-mediated bilayer destabilization, whereas introduction of a photopolymerizable group in the head group region can yield stable liposomes (Figure 3). In the latter liposomes system, bilayer stability is achieved following light-mediated crosslinking before injections *in vivo*. These systems are described below. 

### 6.1. Photo-Stabilized Liposomes as Candidates for Sustained Drug Delivery

Liposomal lipids are known to interact with plasma components causing destabilization of the lipid bilayer liposomes both *in vivo* and *in vitro* [111]. Moreover, soon after the discovery of liposomes as potential drug delivery systems, it was realized that the liposomes were preferentially captured by the reticuloendothelial system (RES). These observations resulted in limited success of liposomes as drug carriers *in vivo* [52,53]. To bypass RES uptake of liposomes, stable liposomes formulations containing lipids containing poly(ethylene glycol) (PEG) in their head group [52,53] are considered suitable systems for drug delivery. Pegylated liposomes are currently the most widely used formulations for *in vivo* applications (reviewed in ref. [2]). An alternate approach to stabilize liposomes utilizes photoreactive lipids with modification in the phospholipid head group by introducing a photopolymerizable group [112,113]. The chemical structure of the head group polymerizable lipid, DPPE-DVBA is shown in Figure 3. The idea to generate plasma stable liposomes using this strategy involves photo-crosslinking (light-induced polymerization) of drug-loaded liposomes under relatively mild conditions. The photo-crosslinking is typically initiated by a water soluble free radical initiator. These molecules contain a 3,5-divinylbenzoyl functionality [112] or *N*-(4-vinylbenzoyl) head group [113]. Liposomes prepared from these lipids have been demonstrated to photo-crosslink in the presence of UV light without compromising the activity of entrapped enzymes [113]. These formulations are attractive candidates for sustained drug delivery; however, practical applications of head-group polymerizable liposomes have not been explored yet. As mentioned earlier, fatty acyl chains are important determinant of liposomal bilayers, several groups have introduced modifications in the fatty acyl chains with the aim to generate liposomes for localized drug delivery. Currently reported phototriggerable liposomes are described below.

### 6.2. Phototriggerable Liposomes for On-Demand Drug Delivery

Phototriggerable liposome drug delivery platforms have been explored since decades and potentially present versatile tunable systems since the features such as the source of wavelength, duration, and intensity of light treatment can be easily adopted as desired. The clinical success of phototriggerable lipid molecules is primarily dependent on two important parameters. First, the photoactivable lipids should retain their liposome forming properties, efficient drug loading and plasma stability traits before phototriggering. Second, the source of light used to activate/destabilize liposomes should be applicable to deep tissue. Moreover, an in depth evaluation of kinetics of drug release from photoactivated liposomes will be instrumental to define the efficiency of treatment modalities. One of the natural phospholipids, plasmalogen was initially studied based on photo-oxidation of its vinyl ether bonds by ROS generated by a suitable oxidizing molecule. A number of phototriggerable synthetic phospholipids are currently available and have been demonstrated to undergo discrete chemical processes including photopolymerization [114], photosensitization [115,116,117,118,119,120,121], photo-isomerization [122], photo-oxidation [68], or the degradation of photocleavable lipids [117,118]. The majority of these lipids undergo light-triggered modifications in conjunction with a photosensitizing molecule either embedded in the liposome membrane or entrapped in the aqueous core. Interestingly, light-induced effects result in irreversible changes in majority of liposome systems with the exception of phospholipid molecules that undergo phototriggering via the *cis*–*trans* isomerization. Mechanisms of light-induced modifications in lipid molecules resulting in drug release have been dealt in a number of recent and previous review articles [13,68,69,70]. Here, I have provided an overview of some of the principles underlying phototriggering mechanisms of various liposome systems. I have also alluded to the recent work from our laboratory on formulations containing a photopolymerizable lipid DC_8,9_PC.

### 6.3. Reversible Phototriggering

It can be envisioned that the programmable nanoparticles with built-in reversible photo-switches are likely to bear merit for regulated release of drug doses with anticipated clinical outcome. Azobenzenes are a class of chemical compounds that undergo photoisomerization of their *cis* and *trans* isomers. These isomers have the properties to undergo reverse isomerization at a particular wavelength [123]. Bisby and colleagues in 1990s designed and synthesized phospholipids containing the azobenzene groups in fatty acyl chains (see Figure 3) [119]. The azobenzene groups undergo *cis–trans* isomerization (420/360 nm) in a wavelength-specific manner resulting in transient/programmed release of entrapped solutes from the liposomes. The validity of this approach was examined using the DPPC liposomes containing a photochromic lipid “Bis-Azo PC” with addition of cholesterol. These liposomes released their cargo upon treatment with visible light in the region of 470 nm. The acyl chains packing was more organized in the *trans* form in these liposomes, which is thermodynamically preferable [120,124]. Another interesting azobenzene lipid molecule, photoisomerizable cholesterol derivatives, was also synthesized by Liu *et al.* [125]. Azobenzene cholesterol derivative also provides the advantage to avoid the spontaneous leakage problem form liposome formulations. Biological application of the two isomerizable liposome formulations mentioned here has not yet been reported. However, since both molecules are responsive to UV/visible wavelengths, their suitability for *in vivo* phototriggering may be challenging. 

### 6.4. Photocleavable Liposomes

Although phospholipids (phosphatidylcholine), the main constituents of plasma membranes of cells, have been the primary focus for design and introduction of photoactivable groups to generate phototriggerable liposomes, other lipid molecules have also been modified to develop phototriggerable liposomes [117,118,126]. For example, the dihydroxybenzophenone-based amphiphiles as the photolabile lipids using the dithiane-based modular approach were reported [126]*.* Similarly, nitropyridine-based self-sensitized photolabile amphiphiles were also synthesized by the same group [126]. Based on biophysical studies, these amphiphiles could be used as the components of conventional liposome formulations for light-triggered release. Srivastava and colleagues synthesized photocleavable amphiphilic lipids to obtain high yields of these molecules with relatively simple steps [117,118]. Their design included inclusion of *O*-nitrobenzyl derivatives as linkers to connect non-polar tails, for example, stearyl amine to polar heads (such as charged amino acids) [117,118]. These molecules were designed on the basis of their susceptibility to light (typically in the UV range) resulting in the breakdown products that can destabilize liposome membrane. As with other available photoactivable lipids, these molecules are also responsive to UV light and hence their *in vivo* application remains a challenging task. 

### 6.5. Photopolymerizable Liposomes

The systems described above utilize membrane perturbations mechanisms either by irreversible modifications (photo-cleavage) of the photoreactive lipids or reversible conformational changes in the lipids (azobenzene derivatives of lipids). During last years, our group has focused on an alternate approach of phototriggering that relies on inter-molecular photo-crosslinking of a diacetylene phospholipid within the liposome bilayer (photopolymerization) rendering the bilayer unstable. It is evident that the segregation of the cross-linking lipid as patches within the liposome bilayer would be critical for this strategy. Previously, another class of photoreactive lipid, bis-sorbyl PC was reported by O’Brien and colleagues to undergo polymerization upon UV light treatment [127]. In the latter case, a photosensitizing molecule preferentially packaged in the liposome bilayer (and in close vicinity to bis-sorbyl PC) was included to activate these lipids in the visible range. I will discuss the properties and drug delivery potential of two photopolymerizable phospholipid molecules namely bis-SorbPC and DC_8,9_PC (Figure 3) developed for light-triggered drug delivery. 

### 6.6. Bis-SorbPC

O’Brien and colleagues pioneered the concept of using UV light-induced photopolymerization bis-sorbyl phosphatidylcholine (bis-SorbPC), a component of liposomes to promote release of liposome-entrapped contents [127,128,129]. In an attempt to develop these liposomes applicable to cellular assays, a cationic lipophilic dye, 1,1'-didodecyl-3,3,3',3'-tetramethylindocarbocyanine perchlorate (DiI, as a hydrophobic photo-sensitizer) was included in these liposomes. DiI containing liposomes, when treated with visible light (550 nm) triggered release of entrapped contents. The photopolymerization initiation of bis-SorbPC is considered to occur via the oxygen radicals, produced by activation of DiI by 550 nm wavelengths. It is clear that packing of lipid and the photosensitizer in concert is crucial for this photopolymerization mechanism. This system bears merit and it is my viewpoint that it will be worth pursuing these platforms by using hydrophobic probes and/or new PDT drugs that can be activated by the near-IR wavelength light sources. We have also reported that the entrapment of a water soluble photosensitizer in liposomes containing a diacetylenic lipid promotes a similar outcome (discussed below). However, the mechanism of destabilization of liposomes containing DC_8,9_PC are unrelated to photopolymerization. We have recently reported that the spectral properties of photosensitizer entrapped in these liposomes plays an important role in the visible light-induced phototriggering. Our studies on DC_8,9_PC formulations are discussed below. 

### 6.7. DC_8,9_PC

The photopolymerizable phospholipid, (1,2-bis(tricosa-10,12-diynoyl)-*sn*-glycero-3-phosphocholine (DC_8_,_9_PC, Figure 3), has been studied since 1980s [114,130]. This lipid uniquely assembles into the lipid bilayer due to the presence of triple bonds in the fatty acyl chains. It is also well-established that DC_8_,_9_PC undergoes UV (254 nm)-induced photopolymerization accompanied by change in its chromogenic properties. Potential biological applications include functionalized polymerized vesicles for vascular-targeted molecular imaging [131], oral vaccine preparations [132], and DNA delivery [133,134]. In our recent review, we have discussed polymeric lipid assemblies and their applications in biology and theranostics in detail [135]. 

**Figure 4:**
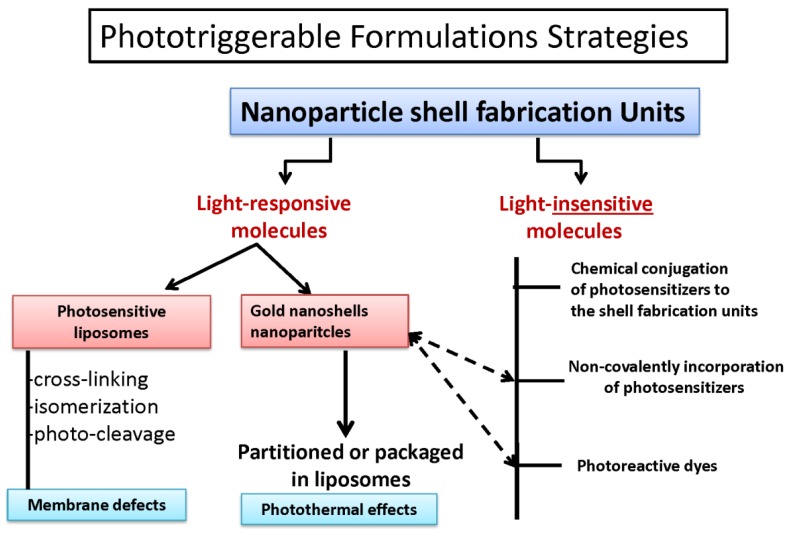
Phototriggerable formulation strategies. A broad scheme of fabrication of liposomal nanoparticle sensitive to light is shown. Liposome core is fabricated either using the light-responsive molecules (such as photosensitive lipids, see Figure 3). Alternatively, non-photoreactive lipids are used to make the liposomes and light-reactive molecules are added to the liposomes using various strategies as indicated.

**Figure 5:**
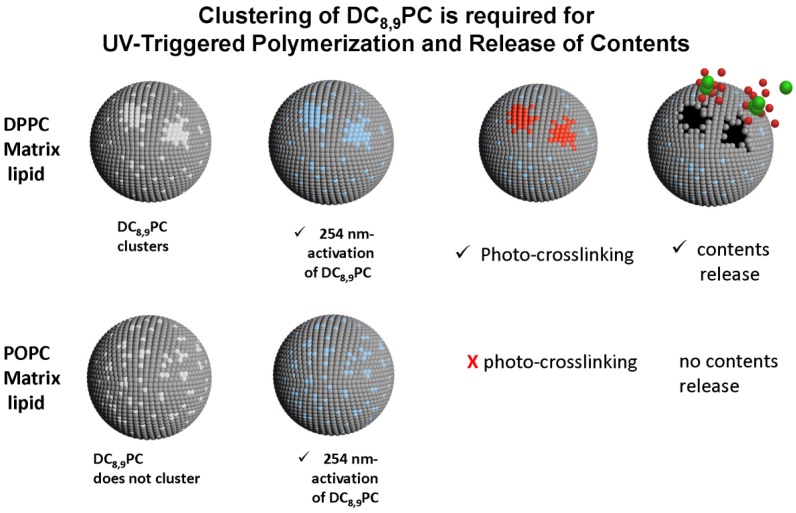
DC_8,9_PC formulations: effect of Matrix Lipids and Phototriggering by UV (254 nm) light. DPPC or POPC as matrix (bulk) lipids (grey, as indicated, DC_8,9_PC, white before light activation, blue after light activation. Cluster, bright red, causing defects in liposomes. Release of entrapped cargo (green, dark red).

We were the first to report the *in situ* light-triggered drug release properties of liposomes containing DC_8,9_PC [121]. We had hypothesized that, DC_8,9_PC is likely to form aggregates (self-assemble) in the bilayer of phospholipids containing saturated acyl chains, and this packing is prone to create phase boundary defects in lipid model membranes (Figure 5). Our experiments included liposomes containing DC_8,9_PC (*T*m ~44 °C) with either a saturated lipid DPPC (*T*m 41 °C) or unsaturated lipid POPC (*T*m ~2 °C) as the matrix lipids. The cartoon (Figure 5) shows matrix lipids as dark grey and DC_8,9_PC as white balls. UV (254 nm) treatment changes the chemistry of DC_8,9_PC (blue balls). However, UV (254 nm)-triggered calcein release occurs only from liposomes containing a mixture of saturated phospholipids and DC_8,9_PC (Figure 5, top panel) with clear evidence of photopolymerization of DC_8,9_PC (shown in red clusters). In contrast, POPC:DC_8,9_PC liposome formulations do not show any evidence of polymerization and hence fail to release calcein. 

**Figure 6:**
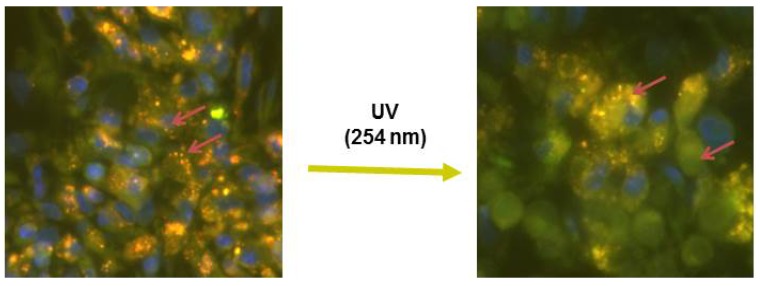
Light-triggered release of liposomal doxorubicin from liposomes in tumor tissue. Liposomes prepared from DPPC:DC_8,9_PC:DIR:folate (89:10:0.5:0.5 mol%)-loaded with doxorubicin were injected in mice with KB Xenografts. Four hours post injections, tumors were taken out and thin sections (OCTs) were prepared. The tissue sections were imaged before (**left**) and after treatment (**right**) with UV (254 nm). Images were collected using the Nikon 80i Microscope, equipped with Andor 885 EM-CCD camera and Sutter Lambda LS light source (Nikon Instruments Inc., Linthicum, MD, USA). Before treatment, liposomes accumulated in tumor area are shown as punctate florescent particles. Following 254 nm treatment, doxorubicin is distributed in a large area as indicated by arrows.

We have further developed DPPC:DC_8,9_PC formulations (loaded with doxorubicin) to demonstrate their potential for light-triggered drug delivery using the *in vitro* [136] as well as cell-based assays. The outcome of these studies yielded some interesting findings. The solute release (doxorubicin or calcein green) occurred upon treatment with a 514 nm laser. In contrast to UV (254 nm)-triggered release from these formulations, 514 nm laser-triggered release was dependent on the fluorescent properties entrapped solute. For example, calcein blue (*E*x/*E*m 360/460 nm) was released by the 254 nm but 514 nm laser treatment. We were not able to detect a clear evidence of photo-crosslinking of DC_8,9_PC upon 514 nm-laser treatment. In contrast, UV (254 nm) treatment results in DC_8,9_PC photo-crosslinking. Moreover, 514 nm-triggered release of doxorubicin resulted in improved cytotoxicity in cell culture system. To our knowledge, we are the first to show the desired effect on cytotoxicity of released drug following phototriggering from a liposome formulation. Although, the exact mechanism(s) by which 514 nm laser treatment destabilizes these formulations and releases entrapped contents are still under investigation, we have recently demonstrated that the 514 nm laser-mediated phototriggering occurs primarily via a Type-I photoreaction process [137]. Therefore, DPPC:DC_8,9_PC formulations are viable candidates for light-triggered drug delivery to treat cancer patients. Current work using these formulations is in progress using mouse models (tumor xenografts) and the initial studies show promise. Although it is premature to comment on our unpublished tumor regression data in this review, I would like to mention that we have successfully demonstrated *ex vivo* liposome destabilization in tumor tissues using the 254 light source (Figure 6).

## 7. Phototriggerable Liposomes for Cancer Treatment: Limitations

As described above, a number of light-triggerable liposome formulations have been examined to date; however, their *in vivo* applications remain to be documented. Majority of designed phototriggerable lipid molecules synthesized thus far are tunable by the light sources in the UV (or visible) range, posing limitations to penetrate into biological tissues. The second limitation may be the lack of adequate photon energy produced by the light sources in the biological tissues. In my opinion, the combination of currently available (and new) photosensitizing drugs with the phototriggerable formulations may be one of the avenues to pursue. Alternatively, innovative approaches to combine metal ions (or other helper components) with currently available photoactivable lipids may provide an opportunity to achieve required photon energy for liposome destabilization. Seminal work by Joshi, Halas and colleagues using the gold nanostructures as theranostics tools presents opportunities to gain insights into the metal-ion based therapies [138,139,140]. Although this area is beyond the scope of this article, further information can be found at http://www.nanospectra.com/technology/aurolasetherapy.html. Similarly, infrared light sources currently in use for PDT should be taken into consideration towards development of phototriggerable liposomes for cancer treatment. 

## 8. Clinical Promise and Challenges towards Future of Phototriggerable Liposomes in Drug Delivery

The field of cancer nanomedicine has progressed in recent years. Spatial and temporal release of nanoparticle-encapsulated drugs (as well as other biomolecules) in a regulated fashion at the site of action is one avenue that calls for attention to impart further improvement in treatment modalities. Therefore, nanoparitcles with built-in tunable triggering properties platforms coupled with localized drug delivery technology will have significant impact on cancer therapy and other related diseases. In the field of liposomes, triggering is achieved by a common mechanism, *i.e.*, perturbation of the liposomal bilayer. Thermosensitive liposomes are the best-studied examples in the field of liposome drug delivery. However, the availability of PDT drugs and our understanding of the photochemical reactions *ex vivo* and *in vivo* provides with a firm platform to develop phototriggerable liposomes suitable for clinical use. Phototriggerable liposome drug delivery systems have been developed for decades. Several of these systems have been demonstrated to be potentially viable either *in vitro* or in cell-based systems. A major challenge remains to demonstrate their suitability *in vivo* for improved drug delivery and/or tumor regression in animals. Recent developments in the laser systems/light guides are important advances that will aid towards development of phototriggerable liposomes. It may be noted that recently reported alternate theranostics platforms, such as carbon dots and PS-functionalized gold nanostars were demonstrated to show promise based on animal studies, In general, the activation mechanism of these nano-systems entails PDT in combination with plasmonic photothermal therapy. These systems are likely to offer an advantage because they are effective in hypoxic conditions. However, the efficiency of drug loading in these particles may be a limiting factor. In my view, the following areas are worth considering towards design and development of clinically suitable liposomes. Previously described phototriggerable lipids (such as bis-sorb PC) could be revisited and explored using new dimensions in combination with recently developed wavelength-specific photosensitizers (PDT drugs), more so the ones which are in the clinics and/or clinical trials. Based on the potential success of Aurolase therapy (gold nanoshells) and other similar metal-based nano-platforms, biocompatible metal ions (such as gold) deserve a closer look as potential activation ingredients. The concept of reversible or irreversible phototriggerable liposomes is currently shaded. Detailed analysis of mechanisms of phototriggering (either burst or programmed) is a critical element to monitor the rate of release of drugs. Also, information about the kinetics and extents of drug release upon phototriggering is warranted. The possibility to tune reversible phototriggerable liposomes for repeat treatments without repeat injections into patients is likely an advantage in the drug delivery field. Lastly, Visudyne therapy presents opportunities and strengthens the future of phototriggerable liposomes. Visudyne therapy is localized for ocular treatment (an area more prone to photosensitization by light and/or environment). It can be predicted that the treatment of organs such as bladder and prostate will have a better outcome by light-triggered drug delivery technology.

## Abbreviations

PC: Phosphatidylcholine
PE: Phosphatidylethanolamine
Tm: Phase-transition
DPPC: 1,2-dipalmitoyl-*sn*-glycero-3-phosphocholine
POPC: 1-palmitoyl,2-oleoyl-*sn*-glycero-3-phosphocholine
DiI: 1,1'-didodecyl-3,3,3',3'-tetramethylindocarbocyanine perchlorate
DC_8,9_PC: Photo-polymerizable phospholipid: 1,2-bis(tricosa-10,12-diynoyl)-*sn*-glycero-3-phosphocholine
PS: Photosensitizer
PDT: Photodynamic therapy
ROS: Reactive oxygen species
EPR: Enhanced Permeability and Retention Effect
